# Therapeutics

**Published:** 1899-12

**Authors:** Bertram M. H. Rogers


### THERAPEUTICS.

**Enteroclysis or irrigation of the large intestine** in the treatment of summer diarrhœa in children does not seem, if one may judge from the amount of literature published on the question in this country, to have been received with the favour that has been accorded to it in America or on the Continent. The most modern text-books published in England mention it, but not in that enthusiastic tone adopted by physicians in the United States. For instance, in the sixth edition of Goodhart[1] the matter is dismissed in eight lines, and Dawson Williams,[2] though advocating it, speaks of the use of "clysters" in summer diarrhœa, and not of irrigation of the bowel, using only a pint of water, which is retained for half an hour. The excellent results which are recorded by American physicians, as well as my own experience, induce me to recommend it strongly in most cases of diarrhœa in children, whether the attack is one of so-called "summer diarrhœa" or due to other causes.

It was at first thought that this line of treatment would be only applicable for those children not severely attacked, and so not collapsed, but experience of the beneficial effects it produces now warrants it in all cases; in fact, the weaker the child, provided it is not moribund, the greater is the indication for enteroclysis.[3] Before all things, it is necessary to remove the toxic matters in the intestines, and no surer or quicker way is obtainable than by washing out the large bowel. Not only is the intestine cleaned, but peristalsis is encouraged, and the warmth of the water stimulates the failing vitality, the fluid, as is well known, supplying fresh liquid to the circulation. It is claimed that cerebral thrombosis, due to the drain upon the blood, is prevented by this means.

By some physicians **lavage of the stomach**, which can very easily be done in quite young children, is recommended as a preliminary to washing out the bowel; but, except in cases where there is much vomiting, it is not necessary, and many cases do quite well when it is omitted. Should it be considered advisable, the child should be placed on its back and a No. 20 French size indiarubber catheter, connected with a tube and funnel, passed down the œsophagus. Normal saline solution is then poured in and withdrawn in the usual manner till the fluid comes away clear. Care must be taken in small children not to over-distend the stomach or insert the fluid at more than a very moderate pressure, as the fluid cannot escape by the side of the tube as it does from the bowel. Jacobi[4] recommends a disinfectant being added to the fluid, such as thymol, 1 in 3,000, or resorcin, 1 in 1000, but this is not necessary. The temperature of the saline solution should be 98.6° F.: but if there is fever it may be below that temperature; if much collapse, above. He also says that pure boiled water should not be employed, as it causes "osmosis of the body fluids into the stomach, sometimes to such an extent as to visibly increase the amount returning from

[1] *Diseases of Children*, 1899, p. 63.
[2] *Medical Diseases of Infancy and Childhood*, 1898, p. 426.
[3] Lockhart Gillespie, *A Manual of Modern Gastric Methods*, 1899, p. 141.
[4] *Therap Gaz.*, 1899, xxiii. 505.

the stomach," which, of course, in any stage of this disease is undesirable. This procedure need seldom be done more than once, whereas irrigation of the bowel may be performed oftener, and in many cases must be.

To **perform enteroclysis** a soft rubber No. 14 English catheter, attached to a fountain syringe, or tube and funnel, is passed just within the sphincter and normal saline solution allowed to run in. In my cases we have always used a tube and funnel, as the pressure can be regulated to a nicety, and we generally raise the funnel about two feet. Jacobi[1] recommends four to twenty inches; Holt[2] as much as four feet, which, though high, does not appear to have done any harm. Lichty[3] says there is no fear of rupture of the bowel, since the rectum will expel the water after a certain pressure is obtained. As the sigmoid flexure becomes distended the catheter may be inserted further, the folds of that part of the bowel becoming opened. How far the fluid will reach will depend on the pressure, but little difficulty will be found in reaching the ileo-cæcal valve or even higher. Records of it reaching the stomach must be received with caution, if not scepticism. Ssokolow[4] points out that over-distension of the rectum will lead to expulsive efforts and prevent the fluid reaching higher parts, and he was able in 103 patients out of 200 experimented on to pass water through the valve. Lavage of the small intestine is not however desired, as the peristalsis produced by the evacuation of the fluid will stimulate the small intestine to expel its contents. When about a pint or thirty ounces has been passed into the bowel of an eighteen months child, Kerley[5] advises that the solution should be allowed to run in and out at the same time, but the tube should not be passed in more than nine inches. I have employed a double catheter in irrigation, stopping up the escape one till I wished to allow the escape to begin. Hubbard[6] breaks the connection with the funnel and catheter when the baby begins to strain, and allows the outflow to pass through the catheter.

The **fluids employed** are: normal saline solution, salt solution 1 in 7,000 (Jacobi), boric acid .5 per cent. (Dawson Williams and Ashby), boiled water (Mercier), HCl solution 1 per cent. (Grimm), sulpho-naphthol (Hubbard); but the first mentioned appears to work quite satisfactorily. Starch solution is recommended if there is much inflammation, and Thomson[7] says that American authors advise leaving in the bowel 15 to 20 grains of tannic acid to render inert soluble peptones. The amount of fluid used is generally about two quarts, but as the excess escapes even larger quantities than that may be used.

[1] *Loc. cit.* [2] *Therap. Gaz.*, 1899, xxiii. 512. [3] *Med. News*, 1898, lxxii. 496.
[4] *Jahrb. f. Kinderh.*, 1894, xxxviii. 186; abstract in *Am. J. M. Sc.*, 1895, cix. 481.
[5] *N. York M. J.*, 1898, lxviii. 145.
[6] *Arch. Pediat.*, 1899, xvi. 263.
[7] *Clinical Examination and Treatment of Sick Children*, 1898, p. 281.

**Irrigation** should be the first means of attack, being employed as soon as the patient is seen, and may be repeated every two hours till all toxic material is removed from the bowel. No purgatives need be given, no astringents, and sedatives such as opium are contra-indicated, as they prevent the peristaltic action of the small intestine which is necessary to push on the materials there. In fact, as Jennings[1] says, "with the thorough use of water internally and externally, opium and other sedative and astringent drugs will rarely be necessary;" while Holt[2] says it is "of more value than anything else we can do for these cases," and Graham[3] describes it as "an absolute necessity," and declares that "many cases are undoubtedly lost through a failure to appreciate their usefulness." In the face of such strong statements as these, it is remarkable how little English physicians appear to have adopted this line of treatment, which is at the same time effective and simple.

BERTRAM M. H. ROGERS.

---

### Reviews of Books.

[1] *Therap. Gaz.*, 1899, xxiii. 589.
[2] *Loc. cit.* [3] *Therap. Gaz.*, 1899, xxiii. 517.

